# Use of individual Google Location History data to identify consumer encounters with food outlets

**DOI:** 10.1186/s12942-025-00387-w

**Published:** 2025-02-15

**Authors:** Olufunso Oje, Ofer Amram, Perry Hystad, Assefaw Gebremedhin, Pablo Monsivais

**Affiliations:** 1 School of Electrical Engineering and Computer Science, Washington State University, Pullman, WA USA; 2 Department of Nutrition and Exercise Physiology, Elson S. Floyd College of Medicine, Washington State University, Spokane, WA USA; 3 College of Health, Oregon State University, Corvallis, Oregon USA

**Keywords:** Geospatial behavioral analysis, Food environment interaction, Dietary pattern mapping

## Abstract

**Background:**

Addressing key behavioral risk factors for chronic diseases, such as diet, requires innovative methods to objectively measure dietary patterns and their upstream determinants, notably the food environment. Although GIS techniques have pushed the boundaries by mapping food outlet availability, they often simplify food access dynamics to the vicinity of home addresses, possibly misclassifying neighborhood effects. Leveraging Google Location History Timeline (GLH) data offers a novel approach to assess long-term patterns of food outlet utilization at an individual level, providing insights into the relationship between food environment interactions, diet quality, and health outcomes.

**Methods:**

We leveraged GLH data previously collected from a sub-set of participants in the Washington State Twin Registry (WSTR). GLH included more than 287 million location records from 357 participants. We developed methods to identify visits to food outlets using outlet-specific buffer zones applied to the InfoUSA data on food outlet locations. This methodology involved the application of minimum and maximum stay durations, along with revisit intervals. We calculated metrics from the GLH data to detect frequency of visits to different food outlet classifications (e.g. grocery stores, fast food, convenience stores) important to health. Several sensitivity analyses were conducted to examine the robustness of our food outlet metrics and to examine visits occurring within 1 and 2.5 km of residential locations.

**Results:**

We identified 156,405 specific food outlet visits for the 357 study participants. 60% were full-service restaurants, 15% limited-service restaurants, and 16% supermarkets. Mean visits per person per month to any food outlet was 12.795. Only 8, 10 and 11% of full-service restaurants, limited-service restaurants, and supermarkets, respectively, occurred within 1 km of residential locations.

**Conclusions:**

GLH data presents a novel method to assess individual-level food utilization behaviors.

## Introduction

The intricate relationship between dietary behaviors and chronic disease risk is an area of increasing concern and study. Traditional methods of assessing dietary intake and behavior, chiefly self-report measures, incorporate biases that limit their reliability [1, 2]. These conventional tools, while foundational in nutritional epidemiology, often grapple with recall bias and the influence of social desirability on self-reporting [3, 4].

Research on upstream drivers of dietary intake, namely food access and availability—often referred to collectively as the food environment—has been significantly advanced by the use of spatial methods, particularly through the application of geographic information systems (GIS) to map and quantify the retail food environment [5–8]. The food environment broadly encompasses the physical presence of food outlets, the type and quality of foods available, and the accessibility of these outlets to consumers [6, 7]. Various measures of the food environment exist in the literature, including proximity measures (e.g., distance to the nearest food outlet) and density measures (count of food outlets within a spatial unit, such as a standardized buffer zone around a residential address or within an administrative geographic area) [6]. The density measure is frequently used to assess food access because it captures the overall availability of food outlets within a given area, reflecting potential exposure to different food environments [8].

However, these methods have been criticized for oversimplifying food access dynamics by primarily analyzing neighborhoods around home addresses [9]. This criticism is further supported by the finding that traditional home-based approaches overestimate the importance of the neighborhood food environment [9]. The use of wearable global positioning systems (GPS) technology in food access research can provide detailed tracking of which food outlets are actually visited by participants [10]. However, this approach has been limited in capturing long-term dietary behaviors, because GPS devices are typically worn for a limited number of days [5]. These limitations highlight the need for a more comprehensive and nuanced approach to understanding food access and dietary intake.

The availability of big data derived from everyday technology use, such as smartphones, has opened new avenues for health geography research [11, 12]. Applications on smartphones often collect geospatial data in order to provide location-relevant information and services. These data provide a continuous, passive stream of geolocation data, offering a novel lens through which long-term patterns of food outlet utilization can be viewed. This has the potential to transform our understanding of food environments and their influence on public health.

Recent studies have effectively utilized anonymized population-level time-position data to analyze urban consumption patterns within the residential neighborhood, demonstrating the practical applications of such data in urban planning and public health [13, 14]. However, a limitation of using such population datasets is their inability to link to detailed individual-level information, such as sociodemographic or health outcomes, which are crucial for more targeted public health interventions. The contribution of our study lies in bridging this gap by correlating individual-level time-position data with specific health and demographic details, thereby offering a deeper insight into the relationship between personal behavior patterns and food environment interactions.

In this study we leverage time location data from participant Google Location History (GLH) to objectively detect and quantify consumer encounters with food outlets over time, which may serve as a proxy for dietary behavior. This methodology paper outlines the data processing, algorithms and metrics developed to derive consumer encounters with food outlets from GLH data. We then compare these measures to conventional measures of food environment based on GIS as well as Google’s own detection of place visits, based on their proprietary methods. Understanding consumer encounters with the retail food environment is a critical step towards developing more accurate measure of diet-related behaviors, which are essential for crafting effective public health interventions and policies.

## Methods

### Data source and collection

#### Participant GLH data

This dataset focused on the time-location data for 357 members of the Washington State Twin Registry (WSTR). The WSTR participants shared their GLH data, which consists of longitude and latitude coordinates, timestamps, and the accuracy of these measurements derived from each participant’s GLH [15]. This feature records the locations that users visit, including the time of the visit and the location’s accuracy. In total, we had access to over 287 million records from this passive data collection method giving us an objective view of the participant movements over time. GLH data has been recognized as a viable source for acquiring individual location information, as evidenced in recent research [15] and a powerful new approach to acquiring environmental exposures and their impacts on public health.

#### Outlets data

Our research utilized INFOUSA/DATA AXLE, a commercial dataset containing a census of food outlets in Washington State, providing details like location coordinates and addresses [16]. This dataset was comprehensive, encompassing a wide range of business types. Specifically, we concentrated on food outlets, filtering them from the broader dataset based on their North American Industry Classification (NAICS) codes. The NAICS codes are a standard for classifying business activities, which helped us organize the food outlets for analysis. This focus on food outlets was guided by the methodology detailed in recent research [2], which utilizes NAICS codes to accurately identify and analyze the food environment. The NAICS code are listed in Appendix A.

### Defining food outlet visits

Our study aimed to quantify consumer visits to food outlets using GLH data by employing a clear and interpretable methodology. GLH data includes classifications of place visits, which encompass food retailers. Although the method for generating these classifications is proprietary and lacks detailed information on accuracy and reliability, the data was still usable for our analysis. While the place visit classification does include visits to a variety of outlets, these are not annotated with NAICS codes or descriptions. Consequently, matching the place visit names to their respective NAICS codes or descriptions would require a manual and potentially error-prone process. Furthermore, approximately 20% of the GLH place visits are represented merely by addresses, without specific names of the places visited, complicating the identification and categorization process even further. Despite these challenges, we utilized the GLH place visit data for comparison with our results.

We constructed a buffer zone around each outlet, delineating a specific area where visits would be counted and analyzed. The initiation of a visit was marked by a participant’s entry into this zone, it’s conclusion by their departure, and a non-return into the buffer zone within a specified time limit. This method relied on setting specific parameters to accurately identify consumer visits. These included the precision of the location data, the dimensions of the buffer zone around each outlet, the duration of time spent to qualify as a visit, and the standardization of these visits across the study. Each parameter was critical for ensuring that the data reflected consumer behavior at these food outlets.

### Food outlet visit parameters

#### Location precision

Accurately identifying visits hinges on the precision of the participant’s location data. Precision is quantified by the accuracy radius around the recorded location point, measured in meters. A smaller radius indicates higher precision. We evaluated the precision of all location points from the GLH data, selecting only those with a sufficiently small accuracy radius for our analysis. This process helped reduce the possibility of falsely identifying visits, ensuring that our analysis was based on reliable and precise location information. In line with these criteria, we used a location precision threshold of 50 m. This threshold was chosen as it strikes a balance between capturing a high number of genuine visits while minimizing the inclusion of erroneous data.

#### Outlet buffer zone dimensions

Determining the appropriate dimensions for outlet buffer zones was crucial in our methodology to accurately identify consumer visits. A larger buffer zone might include a wider area, raising the chance of capturing incidental visits not aimed at the outlet itself. On the other hand, too small a buffer zone could miss genuine visits due to slight inaccuracies in the location data.

To address this, we tailored buffer zone sizes to the types of food outlet, acknowledging that different outlets vary in physical size and attract different patterns of consumer behavior. We established buffer zone dimensions based on the median physical dimensions of outlets in each NAICS category, considering how the size of an outlet might impact the detection of actual visits. This nuanced approach allowed us to balance the need for accuracy with the practicalities of capturing consumer behavior across a variety of food outlet types. Table 1 shows the differentiated outlet buffer sizes for the different outlet categories.

**Table 1:** Visit parameters and their default values

Parameter	Description	Default value	Alternate values
Location Precision	Precision value for each individual GLH point	< 50 m	25 m, 75 m
Outlet Buffer Zone	Circular zone around outlets used to detect a potential visit. Buffer sizes vary based on the median size of each outlet category	Convenience Stores: 25 m Department Stores: 55 m Fruit & Vegetable Markets: 25 m Full-Service Restaurants: 25 m Limited-Service Restaurants: 25 m Supermarkets/Other Grocery (excluding Convenience) Stores: 35 m Warehouse Clubs & Supercenters: 55 m	− 10 m, + 10 m
Minimum Stay Duration	Minimum required time for visit consideration	3 min	1 min, 5 min
Maximum Stay Duration	Maximum required time for visit consideration	3 h	2 h, 4 h
Revisit Interval	Maximum required for consolidation 2 potential visits into one	3 h	2 h, 4 h

#### Visit duration

To accurately detect and classify visits within the buffer zones around food outlets, we defined three essential parameters:

1. *Minimum Stay Duration* This criterion determines the shortest time an individual must spend in the buffer zone to count as a visit. We set this parameter to 3 min [17], helping to distinguish genuine visitors from those merely passing by the location.
2. *Maximum Stay Duration* This limit is set to exclude stays that are likely not indicative of typical patronage, such as employees working at the outlet or individuals using the space for extended periods unrelated to consumption. We set this parameter to 3 h, with stays exceeding this duration not considered valid visits in our analysis. Typically, families spend an average of 53 min at fast food restaurants [18], we set this higher so we could catch longer stays at the outlets.
3. *Revisit Interval* This parameter specifies how soon an individual can return to the buffer zone for it to be considered a continuation of the original visit or a new visit. This parameter was also set to 3 h for accurately consolidating visits, especially in scenarios where a person exists and re-enters the buffer zone within a short period.

These parameters collectively ensure that our method distinguishes between different types of visits accurately, thereby enhancing the precision of our analysis of consumer behavior at food outlets. By setting specific time frames for the Minimum Stay Duration, Maximum Stay Duration, and Revisit Interval, we further refine our approach to accurately identify and analyze consumer interactions with food outlets. Table 1 presents the five key parameters utilized for identifying visits, along with their respective default values.

#### Standardization of visits

To ensure our analysis of outlet visits was both equitable and precise across participants, standardizing the visit data was crucial. This step addressed the variability in the amount of location data available for each individual. Our standardization focused on creating a uniform measure for comparison, specifically the number of active days per participant. Initially, we considered defining active days as the total number of days with recorded location data for a participant. However, this approach has drawbacks due to uneven data coverage across participants, which could skew the analysis of visit frequencies and durations.

**Fig. 1:**
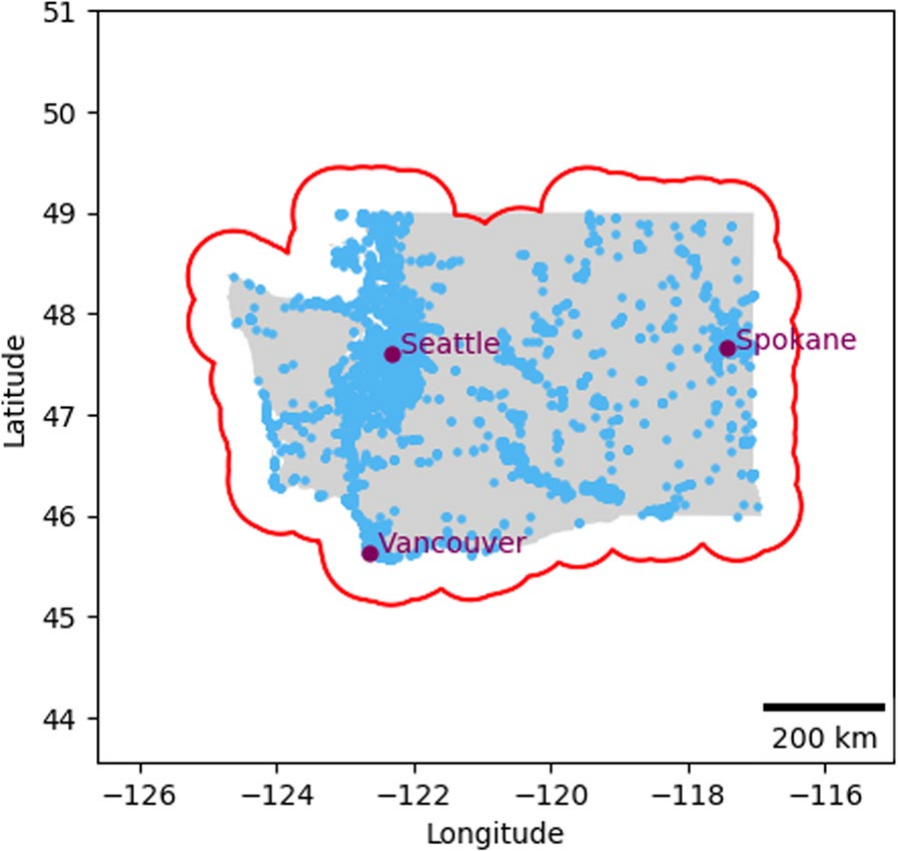
Active area polygon (red) with food outlets (blue) in Washington State (gray). The active area polygon was created using a 50km buffer zone surrounding all outlets

To overcome these challenges, we adopted a more sophisticated strategy. We defined a strategic active area with a 50 km radius around all listed outlets. Fig. 1 shows the delineated active area (red polygon) representing a 50 km buffer zone surrounding all outlets (marked in blue dots). A participant’s active days were then calculated based on their presence within this area. Importantly, we introduced a time-based criterion to enhance accuracy: periods spent outside the active area for more than three hours were not counted as active. This method yielded a more reliable measure of participant activity relevant to our study, ensuring a consistent basis for analyzing visit patterns.

### Data processing workflow

*Initial Filtration of Location Data* The first step in our data processing involves refining the raw location data based on the precision of each location point.

*Construction of Preliminary Visit List* Once the data is filtered for precision, we proceed by isolating location points that fall within the designated buffer zones around each food outlet.

*Consolidation of Visit List* To refine our understanding of visit patterns, we employ the revisit interval parameter. This allows us to merge contiguous visits into a single event, reducing redundancy and providing a clearer picture of consumer behavior.

*Finalization of Visit List* The consolidated list of visits then undergoes a final filtration process. This stage applies the Minimum Stay Duration and Maximum Stay Duration parameters for a visit to be considered valid.

*Standardization of Visits* In the final step, visits are standardized based on the frequency and duration of visits on a daily, weekly, monthly, and yearly basis, considering the time participants spent within the active areas.

**Fig. 2:**
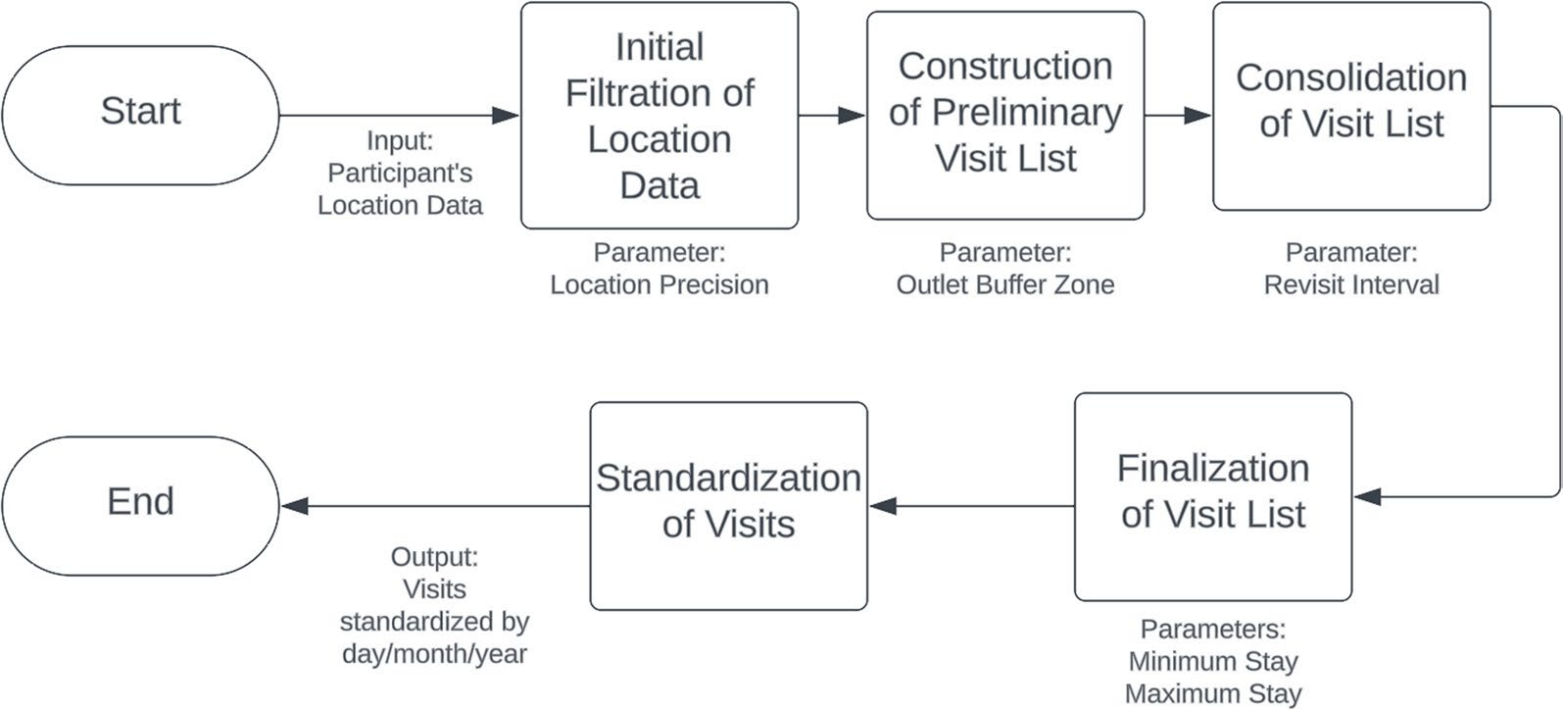
Data Processing Workflow

**Fig. 3:**
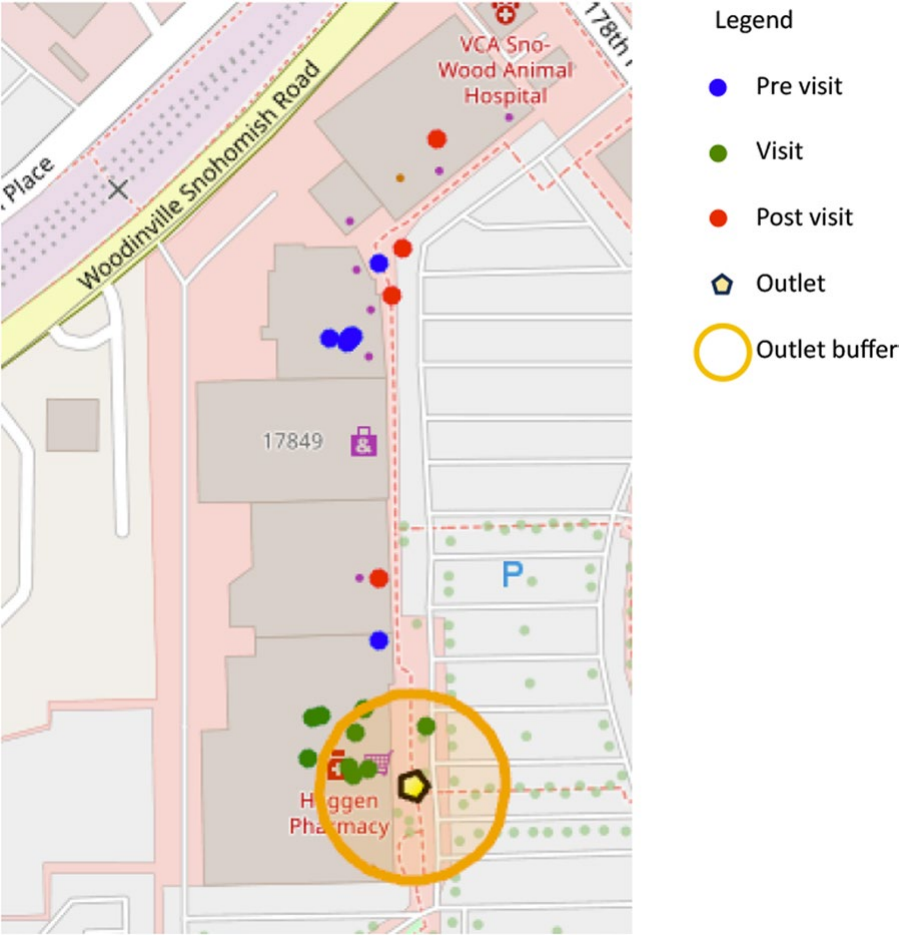
A sample detected visit to a food outlet by one individual participant

Fig. 2 shows the data processing workflow including the parameters used at each stage. Additionally, Fig. 3 illustrates a sample trip trajectory for one individual, showing the applied methodology’s capability to map out visit patterns.

### Sensitivity analysis of visit identification parameters

To assess the impact of individual parameters on the identification of visits to food outlets, we conducted a sensitivity analysis focusing on the five key parameters. This analysis involved systematically altering one parameter at a time-either increasing or decreasing its value-while holding the other four constant. The purpose was to understand how changes in each parameter could affect the total number of visits detected. This method allowed us to determine the relative sensitivity of our visit identification process to each parameter, providing insights into which factors most significantly influence the count of recorded visits. Table 1 shows the parameters alongside their default and alternate values for the sensitivity analysis.

### GIS Comparison and proximity analysis

We derived exposure metrics based on GIS techniques to assess the association between food outlets around participants’ homes and food outlet visits derived from GLH data. Density metrics for supermarkets and limited-service restaurants were computed for distances within 1 km and 2.5 km circular buffers around each participant’s reported residential address. We focused on these two outlet types because supermarkets are primary sources of healthy foods, such as fresh produce and whole grains, which are essential for a balanced diet and have been associated with more favorable health outcomes [6, 8, 19]. Conversely, limited-service restaurants are often associated with the availability of energy-dense, nutrient-poor foods, which have been linked to unhealthy dietary patterns and increased risk of chronic diseases [20, 21]. Additionally, supermarkets are frequently targeted in public health policies aimed at improving food access in underserved communities, making them a key focus for research on the food environment [6, 22, 23]. The 1 km buffer was selected to represent a reasonable walking distance, typically covering 10–15-min walk, as supported by prior studies examining walkability and access to local amenities [5, 24]. The 2.5 km buffer was chosen to reflect a short driving distance, corresponding to a 5 to 7-min drive, aligning with research on vehicular access to food outlets [6, 25]. These considerations guided our decision to focus on these specific metrics.

We counted the number of the outlet types around participants’ homes. Correlation analysis was conducted to determine the relationship between the number of these outlets and visit frequencies. Spearman coefficients were calculated for supermarkets and limited-service restaurants within 1 km and 2.5 km distances. Additionally, we categorized the outlet counts and visit frequencies into four groups (Low, Medium, High, Very High) and computed the Cohen’s Kappa statistic to assess the agreement between outlet proximity and visitation rates.

### GLH Place visits comparison

We analyzed the place visits provided by the GLH dataset, focusing on places within Washington State with an interest in food outlets. Using fuzzy text matching techniques, we aligned GLH data with corresponding NAICS codes and descriptions from our food outlets dataset. Criteria for “location confidence” and “visit confidence” were set at a threshold of 70 or higher, along with a fuzzy text match of 85 or above. The classification results were then compared to those detected by our method to evaluate the robustness and comprehensivenes.

## Results

### Participant characteristics

We collected GLH data from 357 participants in the WSTR. For details on the data collection protocol and a comparison with the broader WSTR cohort, see [15] which discusses our data collection methodologies. Demographic characteristics of the WSTR sample are summarized in Table 2. While these demographics were not used in the analysis, they provide additional information about the sample.

**Table 2:** Sample descriptives

	Male	Female
N	129	228
Age, mean (SD)	46.2 (15.0)	41.9 (14.1)
Income (> $80 k) %	68.2	49.6
Education (≥ Bachelor) %	72.1	73.2
Excellent/Very Good Health %	64.3	58.8
BMI, mean (SD)	26,7 (4.4)	27.1 (6.8)
Area Deprivation Index, mean (SD) *	20.4 (16.6)	27.0 (19.1)
Non-Hispanic White %	89.1	85.1
Married %	69.1	52.2

* Area Deprivation Index from Neighborhood Atlas. For surveys prior to 2016, we used the 2015 national ADI data. For surveys in 2016 and after, we used 2020 national data. Because of the way that it is statistically constructed, the ADI should always only be used in a rank-type format. A percentile splits the ADI scores into 100 equal sections, categorizing the individual block group/neighborhood, with those in the first percentile being the least disadvantaged, and those in the hundredth being the most

### Summary of GLH data

**Fig. 4:**
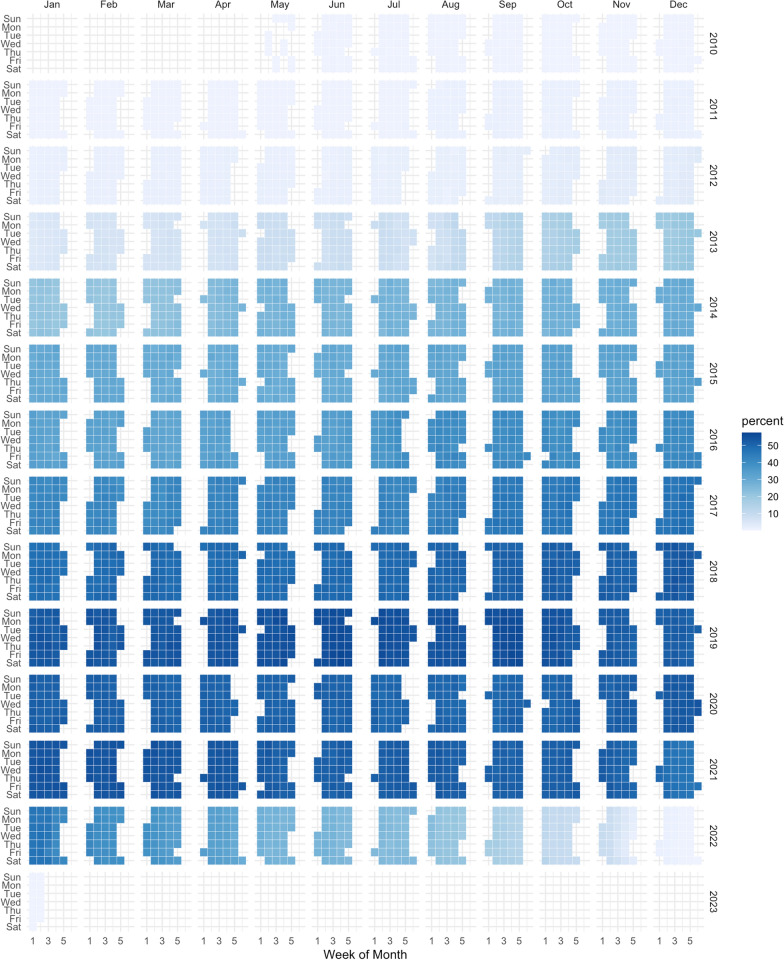
Temporal coverage of data provided by 357 individuals

**Fig. 5:**
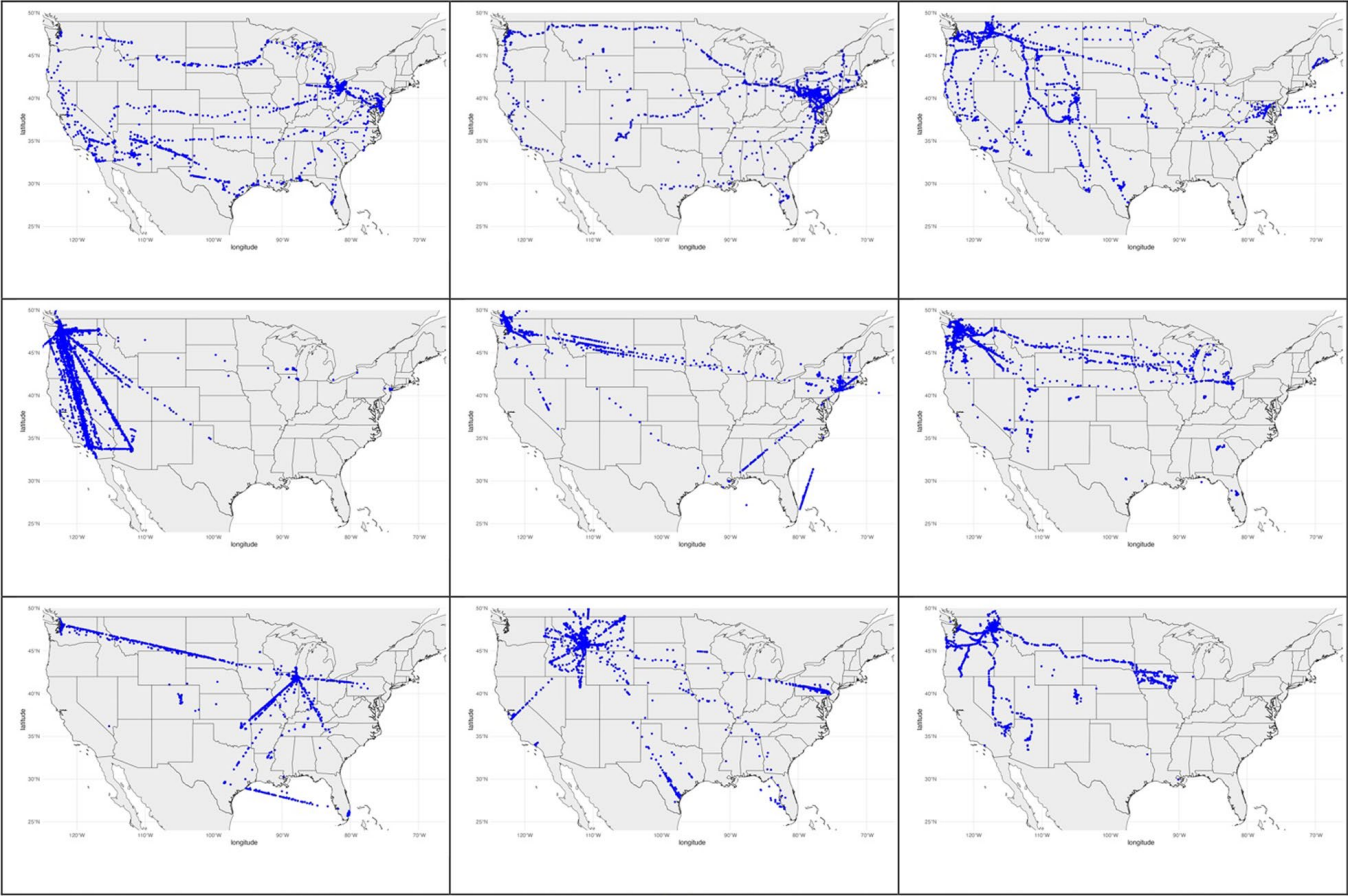
Maps of nine random participants' data ranging from 2012 to 2022

Across all participants, the years over which GLH data were available spanned from 2010 to 2023. This dataset comprises over 287 million raw location records. The duration of data per participants varied substantially, ranging from a minimum of 3 days to a maximum of 3938 days (exceeding 10 years), with a median duration of 1310 days and mean of 1428 days. Fig. 4 presents the distribution of data collection across the years, illustrating the extensive temporal coverage of our dataset. Fig. 5 shows the location data of nine random participants. This figure illustrates the extensive spatial coverage and density of the GLH data.

### Food outlet visit detection

In the application of our algorithm to the GLH data, we identified a total of 156,405 visits to 5098 unique food outlets. The breakdown of visits by outlet type is shown in Table 3. The duration of these trips, encompassing all participants, revealed a mean visit length of approximately 28.58 min. The standard deviation of visit duration was 33.63 min, indicating a broad range of visit lengths. The minimum recorded trip duration was just over 3 min, suggesting brief stops, while the maximum was nearly 180 min, reflective of extended stays. The 25th percentile of trips lasted around 6.35 min, the median was 14.43 min, and the 75th percentile reached 37.2 min.

**Table 3:** Descriptive statistics of food outlet visits for all participants

Outlet type *	Total visits	Median (IQR) of count per person per month	Mean visits per person per month	Mean time spent (h) per person per month
Convenience Stores	1893	0.032 (0.000–0.107)	0.117	0.038
Department Stores	8043	0.175 (0.000–0.600)	0.570	0.205
Fruit & Vegetable Markets	51	0.000 (0.000–0.000)	0.002	0.001
Full-Service Restaurants	93,357	2.962 (1.342–7.511)	8.332	4.879
Limited-Service Restaurants	23,008	0.847 (0.232–1.854)	1.454	0.516
Supermarkets/Other Grocery (excluding Convenience) Stores	25,779	1.070 (0.203–2.519)	2.051	0.688
Warehouse Clubs & Supercenters	4274	0.041 (0.000–0.277)	0.268	0.098
All	156,405	6.632 (2.694–14.640)	12.795	6.425

* Outlet types are categorized based on North American Industry Classification System (NAICS) codes

**Fig. 6:**
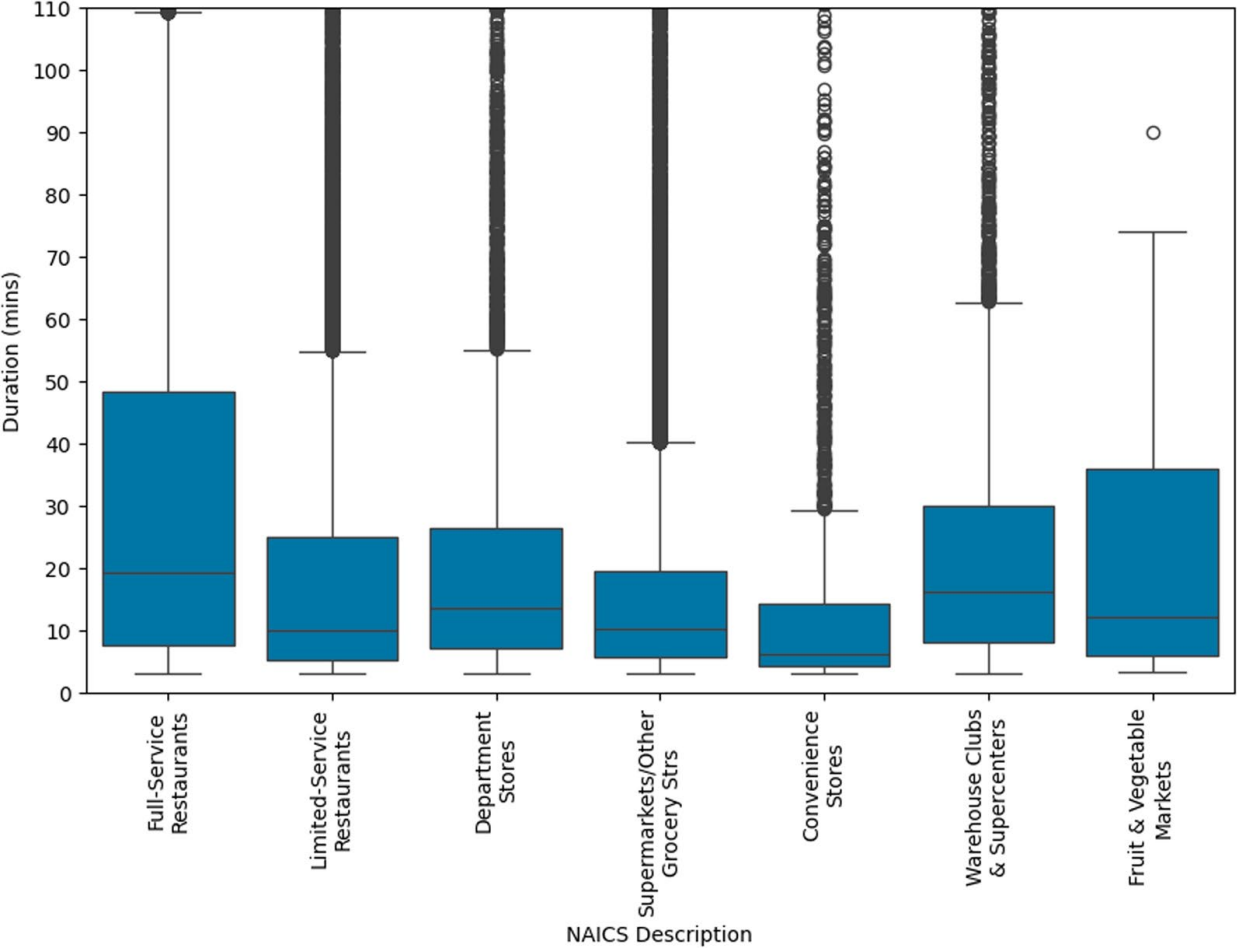
Boxplot distribution of trip durations by outlet category. The figure illustrates the variability and distribution of trip durations across different outlet categories, as defined by the NAICS short descriptions

The distribution of visit durations across different outlet categories is depicted in Fig. 6. The data indicates that participants tend to spend substantially more time at full-service restaurants and fruit and vegetable markets compared to other outlet types. Conversely, convenience stores were associated with the shortest visit duration. This pattern reflects consumer behavior, suggesting that outlets offering sit-down dining or a variety of fresh produce may encourage longer stays, while quick-stop locations like convenience stores typically necessitate less time spent on premises [26, 27].

Out of the 357 participants whose data was analyzed, only 297 had recorded visits. This discrepancy likely stems from instances where participants did not remain within outlet buffer zones long enough to be counted as a visit, or were not present within the active areas predefined for the outlets. Some statistics on the food outlet visits, categorized by outlet type, are provided in Table 3.

### Sensitivity analysis results

**Fig. 7:**
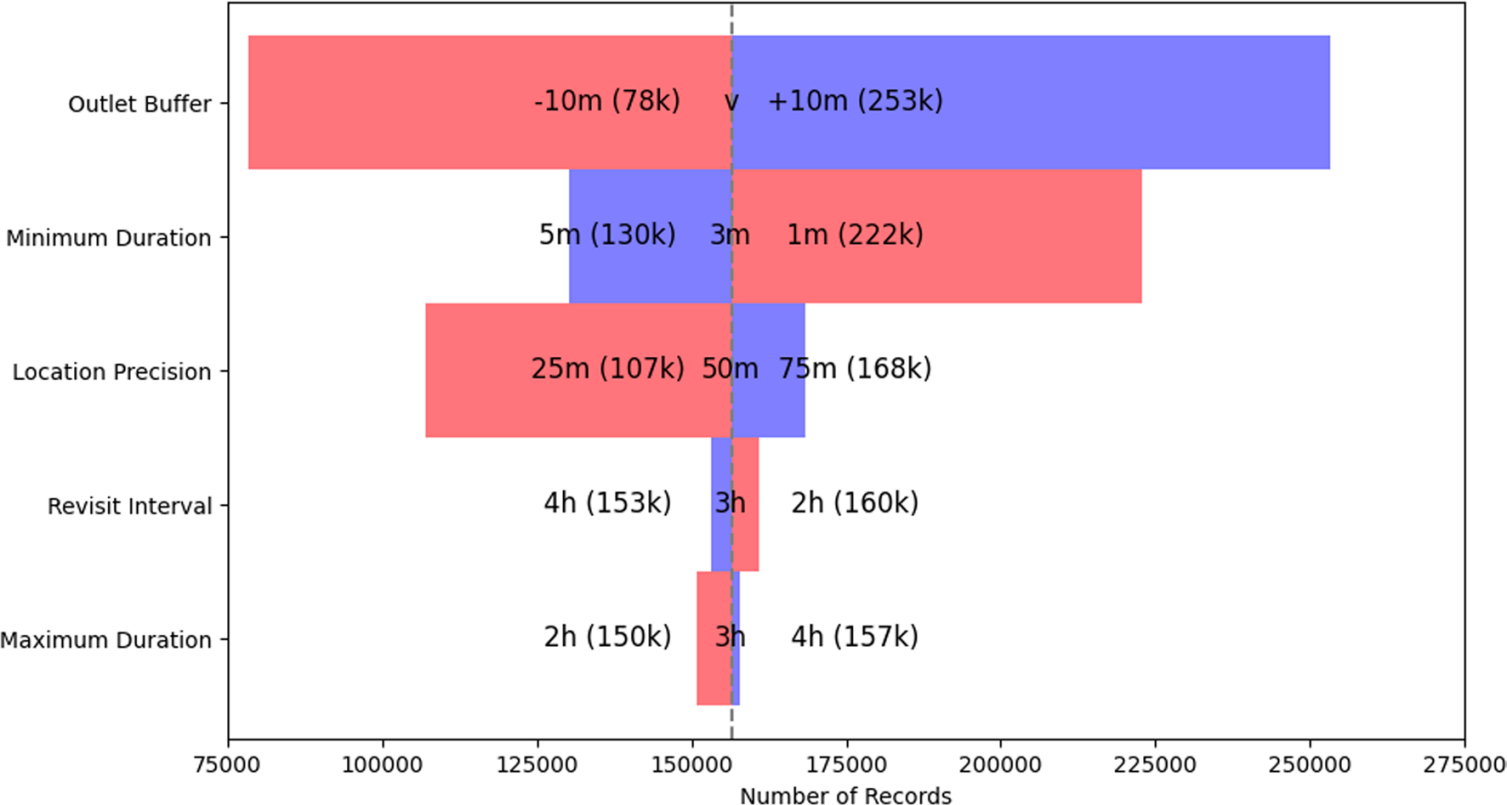
Sensitivity Analysis Plot. This plot visualizes the sensitivity of trip durations to changes in each parameter while holding the other variables constant. Each horizontal bar represents a different parameter, with deviations from a central benchmark value (156,405 records). The red bars indicate the effect of reducing each parameter, while the blue bars show the impact of increasing them. Values annotated on each bar (e.g. ’25 m (107 k)’) represent the specific settings of the parameter and the corresponding number of records affected. For the Outlet Buffer parameter, the central benchmark values are variable (v) and differ for each outlet type (see Methods)

The outcomes of our sensitivity analysis are visually represented in Fig. 7. The analysis revealed that the size of the outlet buffer zone had the most significant impact on the number of detected trips. This is reflected in the data showing a reduction in the buffer zone size by 10 m results in the detection of approximately 78,000 trips, whereas an increase of the same magnitude led to more than 253,000 trips. This effect is logical, as a larger buffer zone encompasses more location points, while a smaller one detects fewer.

Following the outlet buffer in terms of sensitivity was the Minimum Stay Duration. This parameter’s adjustment had a considerable effect on trip detection: decreasing the minimum stay to 1 min identified more than 222,000 trips, while extending it to 5 min resulted in more than 130,000 trips. The sensitivity here can be attributed to the fact that a shorter minimum stay duration naturally detects more brief stops as visits, while a longer duration sets a stricter threshold, thus identifying fewer trips.

Location Precision also demonstrated moderate sensitivity. Altering this value affected the inclusion of raw location data: a more stringent precision threshold of 25 m recognized more than 107,000 trips, whereas a less restrictive threshold of 75 m yielded more than 168,000 trips. The choice of the location precision threshold, therefore, is a trade-off between excluding potential noise and capturing a greater number of visits.

The sensitivity analysis showed that the remaining parameters—Revisit Interval and Maximum Stay Duration–exerted minimal influence on their own. This may suggest an interrelation between these parameters; for instance, setting a Maximum Stay Duration at 3 h would likely detect the majority of visits regardless of a longer Revisit Interval, indicating a saturation point beyond which increasing the interval has little to no effect on visit detection.

### GIS Comparison and proximity analysis

This analysis revealed distinct patterns in visit frequencies relative to the density of outlets around participants’ residences. Visits to convenience stores, department stores, and fruit & vegetable markets showed an increase when comparing the 1 km radius to the 2.5 km radius. In contrast, visits to full-service restaurants and limited-service restaurants were less frequent closer to home but increased within the 2.5 km radius. Table 4 provides the percentage of detected visits within 1 km and 2.5 km of participants’ homes.

**Table 4:** Percentage of detected visits within 1 km and 2.5 km of participant’s home

	Percentage of detected visits	
	Within 1 km %	Within 2.5 km %
Convenience Stores	14	30
Department Stores	15	32
Fruit & Vegetable Markets	22	39
Full-service Restaurants	8	22
Limited-service Restaurants	10	20

The correlation analysis between the number of these outlets and visit frequencies underscored a significant positive correlation, with Spearman coefficients of 0.58 for supermarkets within 1 km (p-value: < 0.0001) and 0.58 for limited-service restaurants within the same distance (p-value: < 0.0001). This positive correlation persisted but slightly diminished in strength at the 2.5 km distance. Table 5 shows the result of these analyses.

**Table 5:** Comparison of supermarket and limited-service restaurant detected visits to GIS-based exposures based on two buffer sizes

	Spearman coefficient	Spearman p-value	Cohen's Kappa	Cohen's Kappa p-value
Supermarkets (within 1 km)	0.577	< 0.001	0.049	0.310
Supermarkets (within 2.5 km)	0.521	< 0.001	0.105	0.028
Limited-Service Restaurants (within 1 km)	0.584	< 0.001	0.105	0.012
Limited-Service Restaurants (within 2.5 km)	0.457	< 0.001	0.138	0.005

Expanding our analysis, we categorized the outlet counts and visit frequencies into four groups (Low, Medium, High, Very High) and computed the Cohen’s Kappa statistic to assess the agreement between outlet proximity and visitation rates. The kappa statistics indicated a generally low to moderate agreement, with the highest observed for limited-service restaurants within a 2.5 km radius (kappa: 0.137, p-value: 0.005). The proximity and correlation analysis using GIS techniques enriches our understanding of how the physical availability of food outlets influences consumer visit patterns, highlighting the complex interplay between accessibility and dietary behavior.

### GLH Place visits comparison

We analyzed the place visits provided by the GLH dataset. Our focus was primarily on places within Washington State, with an interest in food outlets. By employing fuzzy text matching techniques, we aligned GLH data with corresponding NIACS codes and descriptions from our food outlets dataset. Not all GLH place visits are related to food outlets; however, using criteria of “*location confidence”* and “*visit confidence”* parameters set at a threshold of 70 or higher, along with a fuzzy text match score of 85 or above, we successfully matched 102,383 out of 1.9 million records. It is important to note that the exact meanings and distinctions between “*location confidence*” and “*visit confidence*” remain unclear, as these are proprietary metrics defined by Google.

Table 6 outlines the classification results, showing significant variations between the counts from GLH and those detected by our method. For instance, our algorithm identified 93,357 visits to full-service restaurants, substantially higher than the 27,220 reported by GLH, suggesting a more comprehensive detection of such outlets. Conversely, department stores and warehouse clubs & supercenters showed fewer visits compared to GLH, with differences of − 16,102 and − 4718, respectively. These discrepancies might indicate varying sensitivities in the methods to different outlet types or potential underreporting in the GLH data.

**Table 6:** GLH semantic place visit classification results

Outlet Type	Visits reported in GLH data	Visits counted by our method	Difference in count (% difference)	Visits detected by both GLH and our method	Visits not detected by GLH	Detection rate relative to GLH count %
Convenience Stores	1478	1893	+ 415 (+ 28)	49	1845	2.59
Department Stores	24,145	8043	− 16,102 (− 67)	171	7873	2.13
Fruit & Vegetable Markets	39	51	+ 12 (+ 30)	4	47	7.84
Full-Service Restaurants	27,220	93,357	+ 66,137 (+ 242)	7,918	85,454	8.48
Limited-Service Restaurants	9127	23,008	+ 13,881 (+ 152)	212	22,796	0.92
Supermarkets/Other Grocery (excluding Convenience) Stores	31,382	25,779	− 5603 (− 17)	967	24,823	3.75
Warehouse Clubs & Supercenters	8992	4274	− 4718 (− 52)	21	4253	0.49
All	102,383	156,405	+ 54,022 (+ 52)	9342	147,091	5.97

Furthermore, the application of fuzzy matching has its challenges, particularly when the score falls below 90. For example, “Larry’s Drive In” and “Jerry’s Drive In” achieved a match score of 88 despite being distinctly different entities, illustrating a potential for overcounting in our matched records. Overall, our method appears to provide a more robust detection of food outlets visits compared to the GLH data, potentially offering more reliable insights for studies targeting dietary behaviors and public health interventions.

## Discussion

### Interpretation of visit patterns

In our study, the novel application of mobile phone location-time data revealed distinct patterns in the duration and frequency of visits to a variety of different food outlet types. Notably, participants spent more time at full-service restaurants and fruit and vegetable markets compared to other categories. This could suggest a preference for venues offering sit-down meals or fresh produce, potentially reflecting a more health-conscious or social dining behavior. Conversely, the shorter duration at convenience stores might indicate the utilitarian nature of these visits, typically for quick purchases.

Our findings resonate with recent research highlighted in a study which explored the’15-min city’ model using GPS data from 40 million US mobile devices. This study found that the median resident makes only 14% of daily consumption trips locally, emphasizing the significant role local accessibility plays in consumer behavior and potentially dietary choices [13]. The concept of local food access aligns with our observations of frequent visits to nearby full-service restaurants and markets, suggesting a potential leverage point for public health interventions aimed at promoting healthier eating habits.

Furthermore, the popularity of full-service restaurants (93 k visits) over limited-service (23 k visits) ones could inform future research into the quality of dietary intake at different dining establishments. The data also provides a window into consumer behavior, which, when combined with dietary intake data, could lead to more comprehensive insights into how the food environment impacts health outcomes. These findings underscore the need for a multidimensional approach when examining the role of the food environment in dietary choices, one that considers not only the type of food outlets available but also the context and duration of visits to these venues.

### Comparison with traditional data collection

The traditional approach to collecting dietary intake data has often utilized the GIS technique, focusing on spatial analysis of the environments surrounding a participant’s residence [5, 6]. This method has been instrumental in nutritional epidemiology but has faced criticism for potentially oversimplifying the dynamics of food access [9]. The critique centers around its emphasis on residential neighborhoods, which might lead to an overestimated impact of the local food environment on dietary behaviors. Additionally, GIS’s capability to depict long-term dietary patterns is limited, emphasizing the complexity of accurately evaluating food access. This situation highlights the need for methodologies that more intricately capture the nuanced interactions individuals have with their food environments.

The GLH data circumvents the typical inaccuracies associated with self-reporting by providing a timestamped log of user locations, allowing for a more precise reconstruction of an individual’s food outlet visitation patterns. This passive data collection eliminates the potential for recall errors, providing continuous, objective record of individual’s movements over extended periods. Furthermore, the ability to capture large volumes of data may reveal subtleties and patterns that are not discernible through traditional methods.

Furthermore, the integration of GIS techniques to analyze proximity and visit patterns adds a layer of spatial analysis absent from traditional dietary assessment methods. Our GIS comparison revealed significant correlations between the proximity of food outlets to participant homes and the frequency of visits, offering insights into environmental factors that influence dietary choices. Such spatial analyses, grounded in objective data, provide a more nuanced understanding of how the food environment affects dietary behaviors, extending beyond the capabilities of FFQs and dietary recalls.

While the GLH data significantly enhances the objectivity and granularity of our analysis, it is important to acknowledge its limitations. The data, devoid of qualitative dietary intake information, presents an incomplete picture of diet-related behaviors. Therefore, to fully unravel the complex interplay between diet, environment, and health, GLH data should be viewed as complementary to, rather than a replacement for, traditional data collection methods. Combining passive location tracking with dietary intake data promises a comprehensive approach to understanding and improving public health nutrition.

### Limitations and future research

#### Geographical limitation

We focused on food outlets within Washington State, which inherently limits the generalizability of our findings to this specific geographic area. The visit patterns and consumer behaviors observed may not be representative of other regions with different cultural, socioeconomic, and urban planning landscapes. Future research could expand the scope to include a more diverse set of locations, providing a more comprehensive understanding of food outlet utilization patterns across various contexts.

#### Temporal limitation of outlet data

Another limitation pertains to the static nature of our food outlet dataset. The outlet data represents a snapshot in time, lacking the temporal dimension that would inform us about the establishment’s operational status during the data collection period. This limitation may affect the accuracy of visit detection, as we could not discern whether visits correspond to active food outlets or to those that had not yet opened, had already closed, or changed use [28, 29]. Future iterations of this research would benefit from dynamic outlet data, incorporating the opening and closing dates of establishments to refine visit attributions more precisely.

#### Ambiguities in outlet locations

Additionally, we encountered instances where multiple outlets were reported to have the same geographical coordinates, effectively rendering them indistinguishable in our analysis. For example, two different food outlets reported with the same latitude and longitude present a challenge in definitively determining which outlet a participant visited. Although this issue affected only a small fraction of the data, it introduces a level of uncertainty that must be acknowledged. This overlap may occur in complex commercial areas where multiple establishments are housed within a single building or mall. In such instances, our method could not differentiate between visits to adjacent outlets, especially when they fall into different categories.

#### Policy changes impacting data availability

Recent changes to Google’s Location History policy, effective December 2024, introduce limitations for research reliant on this data. Location data will now be stored locally on users’ devices rather than in the cloud, with an option for users to back up this data to the cloud with end-to-end encryption [30]. Additionally, the default auto-delete setting for location data will change from 18 months to three months [30], further restricting the availability of historical data. These changes mean that users may no longer be able to export their location history data from the cloud unless they opt to back it up manually. As a result, the amount of data accessible for studies, such as ours, may be significantly reduced, necessitating alternative methods for data collection or adjustments in study design to accommodate these limitations.

#### Future directions

To mitigate the ambiguity in outlet location data, future research should aim to refine spatial data processing techniques. Incorporating additional data layers, such as zoning information or detailed commercial property maps, could help distinguish between outlets reported at the same coordinates. Moving beyond the conventional method of using a point and buffer for trip detection, an innovative approach would involve utilizing the actual building footprint and extruding around the building, which may offer a more precise identification of consumer visits to specific locations. More sophisticated algorithms that consider the three-dimensional layout of commercial spaces could potentially improve visit attribution accuracy. Moreover, examining the temporal dynamics of food outlet operations may provide insight into consumer behavior patterns about the evolving food environment. This would offer a more detailed understanding of how food outlet availability affects dietary preferences and general health.

Additionally, while our current study focuses primarily on methodological aspects, future research should explore the potential influence of the built environment on food choices and diet more directly. Understanding the complex relationship between food outlet availability, consumer behavior, and health outcomes remains cruicial. Future studies could integrate our data with detailed dietary intake information to better assess how specific elements of the built environment may shape dietary choices and contribute to public health outcomes.

### Policy, planning, and health geography implications

Our study’s findings have significant implications for public health policy, urban planning, and the broader field of health geography. By leveraging GLH data to analyze visit patterns to food outlets, we’ve provided a novel lens through which the interplay between dietary behavior and the food environment can be examined. This approach not only highlights the potential for technology-driven data collection in enhancing our understanding of health behaviors but also underscores the importance of considering geographical context in public health initiatives.

For policymakers, the detailed insights into consumer behavior at various types of food outlets can inform targeted interventions aimed at promoting healthier dietary choices. Understanding that people spend more time at full-service restaurants and fruits and vegetable markets, for example, suggests opportunities for policies that encourage the availability and accessibility of healthy food options in these settings.

From an urban planning perspective, our analysis can guide the development of community layouts that support healthy eating habits. By identifying areas with high concentrations of fast-food outlets versus those with access to fresh produce markets, planners can work to balance the food environment, potentially incorporating zooming regulations that encourage a diverse mix of food outlet types.

In the realm of health geography, this research contributes to a growing body of work focused on the spatial dimensions of health and wellness. It reinforces the need for multidisciplinary approaches that combine geographical analysis with public health and nutrition research, offering a more holistic view of the factors that influence dietary patterns. The methodological advancements presented in this study pave the way for future research that further explores the complex relationships between people, place, and health, potentially incorporating more dynamic data sources and sophisticated spatial analysis techniques.

## Conclusion

This study has demonstrated the utility of GLH data in mapping and analyzing consumer visits to food outlets, offering significant advancements in our understanding of the food environment’s impact on dietary behavior. By employing a refined methodology that includes precise location data filtration, strategic buffer zone designation, and detailed sensitivity analysis, we have provided a comprehensive overview of visit patterns to various types of food outlets within Washington State. Our findings underscore the potential for leveraging passive data collection methods to gain insights into public health concerns, specifically dietary habits, and their environmental determinants.

Moreover, the sensitivity analysis revealed crucial aspects of our methodology that are most influential in visit detection, informing future research directions for improving data accuracy and reliability. The study’s implications extend beyond academic inquiry, suggesting practical applications for public health policy, urban planning, and the enhancement of health geography frameworks. As we move forward, it is clear that integrating geospatial data with heath research offers a powerful tool for addressing complex health challenges, advocating for a more informed approach to promoting healthier dietary behaviors within communities.

In conclusion, this research contributes valuable methodological insights, particularly in understanding how the built environment infuences patterns of food outlet utilization. While not directly examining dietary choices, the findings underscore the importance of exploring the dynamic relationship between place and health. This work encourages continued interdisciplinary collaboration to foster environments that support healthy living.

## Data Availability

No datasets were generated or analysed during the current study.

## Appendix

Appendix A: NAICS Code and their Description

**Table:**

NAICS CODE	NIACS description	N
44512001	Convenience Stores	449
45221001, 45221003	Department Stores	158
44523001, 44523002, 44523003, 44523005	Fruit & Vegetable Markets	14
72251112, 72251115, 72251117, 72251118	Full-Service Restaurants	8577
72251301, 72251302, 72251303, 72251304	Limited-Service Restaurants	2559
44511001, 44511002, 44511003, 44511006	Supermarkets/Other Grocery (excluding Convenience) Stores	1460
45231101	Warehouse Clubs & Supercenters	32
Total Outlets		13,249
